# Detection of Atrial Fibrillation Episodes in Long-Term Heart Rhythm Signals Using a Support Vector Machine

**DOI:** 10.3390/s20030765

**Published:** 2020-01-30

**Authors:** Robert Czabanski, Krzysztof Horoba, Janusz Wrobel, Adam Matonia, Radek Martinek, Tomasz Kupka, Michal Jezewski, Radana Kahankova, Janusz Jezewski, Jacek M. Leski

**Affiliations:** 1 Department of Cybernetics, Nanotechnology and Data Processing, Silesian University of Technology, PL44100 Gliwice, Poland; robert.czabanski@polsl.pl (R.C.); michal.jezewski@polsl.pl (M.J.);; 2 Łukasiewicz Research Network–Institute of Medical Technology and Equipment, PL 41800 Zabrze, Poland; januszw@itam.zabrze.pl (J.W.); adamm@itam.zabrze.pl (A.M.); tomekk@itam.zabrze.pl (T.K.); janusz.jezewski@itam.zabrze.pl (J.J.); 3 Department of Cybernetics and Biomedical Engineering, VSB–Technical University of Ostrava, 708 00 Ostrava-Poruba, Czech Republic; radek.martinek@vsb.cz (R.M.); radana.kahankova@vsb.cz (R.K.)

**Keywords:** support vector machine (SVM), heart rate variability (HRV), HRV features, atrial fibrillation (AF), AF detection

## Abstract

Atrial fibrillation (AF) is a serious heart arrhythmia leading to a significant increase of the risk for occurrence of ischemic stroke. Clinically, the AF episode is recognized in an electrocardiogram. However, detection of asymptomatic AF, which requires a long-term monitoring, is more efficient when based on irregularity of beat-to-beat intervals estimated by the heart rate (HR) features. Automated classification of heartbeats into AF and non-AF by means of the Lagrangian Support Vector Machine has been proposed. The classifier input vector consisted of sixteen features, including four coefficients very sensitive to beat-to-beat heart changes, taken from the fetal heart rate analysis in perinatal medicine. Effectiveness of the proposed classifier has been verified on the MIT-BIH Atrial Fibrillation Database. Designing of the LSVM classifier using very large number of feature vectors requires extreme computational efforts. Therefore, an original approach has been proposed to determine a training set of the smallest possible size that still would guarantee a high quality of AF detection. It enables to obtain satisfactory results using only 1.39% of all heartbeats as the training data. Post-processing stage based on aggregation of classified heartbeats into AF episodes has been applied to provide more reliable information on patient risk. Results obtained during the testing phase showed the sensitivity of 98.94%, positive predictive value of 98.39%, and classification accuracy of 98.86%.

## 1. Introduction

Atrial fibrillation (AF) is the most common heart arrhythmia, which occurs when the atria contracts quickly and irregularly at rates of 400 to 600 per minute. These contractions are independent from ventricles, which themselves operate at much lower rate. AF symptoms often include palpitations, irregular heartbeat, shortness of breath, chest pains and others, but they can be also asymptomatic and is then called silent AF. The frequency of AF occurrence is strictly correlated with the patient’s age [1,2]. The prognosis indicates that the AF occurrence within the period of the next 20–30 years will double, mainly due to the longer life span of the population. The AF detection is important, since this heart arrhythmia is a well-known risk factor for occurrence of ischemic stroke, even six times higher than among patients without the arrhythmia [3].

**Figure 1:**
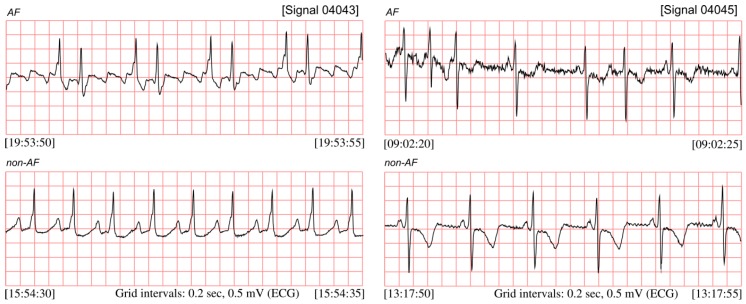
ECG signals (04043 and 04045) taken from the MIT-BIH AF database, with recognized segments of atrial fibrillation and the non-AF ones.

Figure 1 presents the ECG signals from the MIT-BIH Atrial Fibrillation database (MIT-BIH AF) published on PhysioNet [4,5,6], comprising both segments with the AF episodes and non-AF segments. AF episodes occur irregularly and may last from a few heartbeats to hours, which significantly hinder the possibility to diagnose the silent AF by means of occasionally performed ambulatory ECG recordings. It implies that the longer the recording, the higher chance to detect the silent AF episodes [7,8]. The most efficient techniques of long-term monitoring are: Holter monitor, continuous telemetry [9,10,11,12], or implementable devices with internal memory [13,14,15]. However, visual analysis of long 24-h recording requires a lot of time and efforts from the cardiologists, thus the methods for automated detection of atrial fibrillation are needed to improve the objectivity of interpretation. When based on ECG, the efficient automated AF detection requires a high quality signal. It may not be ensured by the long-term monitoring techniques which usually comprise periods of daily physical activity of the patient which distort the ECG signal.

**Figure 2:**
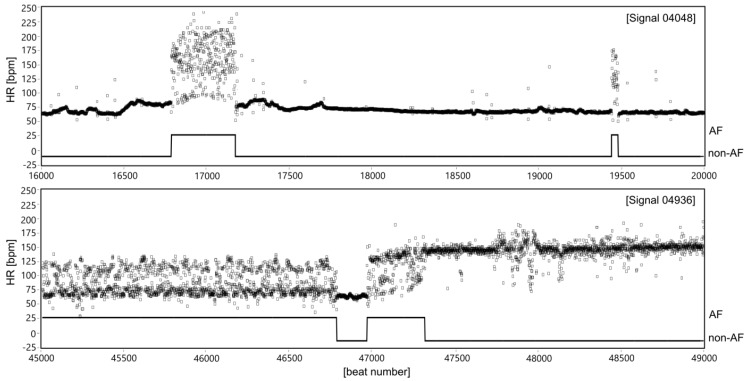
Two HR signals expressed in beats per minute (bpm) with clinically recognized AF segments of different characteristics of HR changes in relation to normal sinus rhythm (non-AF). The AF segments are marked using the experts’ annotations provided for particular records in the MIT-BIH AF database.

The AF episode is manifested in ECG by significant changes of duration of the beat-to-beat (RR) intervals [16,17,18,19], see Figure 1. However, the RR intervals irregularity caused by AF occurrences is much more easy to observe after converting RR intervals into the instantaneous heart rate (HR) signal (Figure 2). The presented HR signals confirm that AF episodes occur very accidentally, and they can last a few seconds (signal 04048), but also expand to long lasting episodes (signal 04936).

In the light of above facts an efficient automated method for AF detection should be based on estimation of RR irregularity or equivalent i.e., HR irregularity observed in long-term recording [20,21]. Moreover, such approach enables to involve the various recording methods which can provide signals in which the heartbeats can be detected. Beside electrocardiogram, such signals include photoplethysmogram [22,23,24] or seismocardiogram [25]. Using a photoelectric sensor is attractive in case of home telecare as long-term recording should be accomplished by instrumentations being minimally troublesome and inconvenient to the patient [26,27]. It may be a smart monitor in a form of a wrist bracelet with a specialized reflective optical sensor to perform the heart rate monitoring using the method previously developed by the authors [28].

The general concept of the methods most commonly used for automated detection of AF episodes relies on determination of features estimating the RR interval changes, and then application of the statistical analysis or more advanced classifier to differentiate between AF episode and normal sinus rhythm segments, basing on the information on RR irregularity. The feature set is composed most commonly of different statistical measures (mean or median HR, root mean square of successive RR differences, turning point ratio). It can also include normalized RR intervals [29,30] or normalized RR differences [31], Shannon entropy [19] or coefficient of sample entropy [15,32]. Other form to present the RR irregularity are: the density histogram of the difference between successive RR intervals [33,34], map that plots RR intervals versus change of RR intervals [35], mapping the RR-interval time series to binary symbolic sequences [36,37] or Markov score of RR interval [16].

In the simplest approach to AF classification the Receiver Operating Characteristics (ROC) curve has been used to find the optimal threshold values for the input features providing the best classification performance [30,35,36,38]. The statistical test (Kolmogorov-Smirnov) was used in [33] to check if the density histograms of the test data differ from the standard density ones prepared as a template of AF episodes. In order to differentiate between AF and non-AF patterns the various classification methods have been applied: Neyman-Pearson detector [31], Random Forest (RF) model and k-nearest neighbors classifier [32], Support Vector Machine (SVM) with promising results reported in [39,40,41], as well as artificial neural network [42], also with interval transition matrices as an input [43].

In [39] SVM approach was used for classification of the 30-s segments of ECG and 300-beat sequences of RR intervals. Two parameters of Stationary Wavelet Transform (peak-to-average power ratio and log-energy entropy) were used for raw ECG-based approach, while five features were extracted from HR signal. The efficiency of AF detection achieved by the feature-based classification of the RR sequences was tested against the algorithm based on raw ECG. Higher sensitivity was ensured by the HR-based approach, while ECG-based algorithm provided improved specificity and classification accuracy. The classifier based on SVM with radial basis function was proposed in [40], with two features as the inputs: the average of RR differences and the standard deviation of differences in a defined duration. The same SVM classifier was employed in [41]. The input set comprised more RR interval features: median heart rate, minimum RR interval, mean RR interval, various entropy measures, and difference irregularity measure.

The features estimating the RR variability are calculated in a sliding window comprising an established number of consecutive RR intervals (or HR values). Since there is no standard for the window length, many works have aimed to find the optimal length, providing the best classification performance. Some works assumed that AF episodes of less than 30 s duration are not clinically significant, which led to higher optimal number of heartbeats: 100 [33], 128 [35] and 150 [37]. Other authors claim that longer windows tend to miss short AF episodes, and thus they applied significantly shorter windows: 30 [32], 12 [15] or even 8 beats [44]. It is obvious that different window length reported as the optimal value depends on the method used for automated AF detection. Another important aspect of using the sliding window for AF detection is how many beats it is shifted. Shifting the window every heartbeat results in one beat resolution of the AF classification. Then each heartbeat (RR interval), usually corresponding to the middle of the window, is classified as AF or non-AF. In such case, determination of classification performance is evident as each automatically classified beat can be related to the reference one, basing on the expert annotations. Otherwise, additional condition has to be applied—the window is labeled as AF episode only if the number of clinically annotated AF beats within the window exceeds a predefined threshold, usually 0.5 like in [35,45]. However, it is obvious that the threshold value affects the classification performance. The threshold has been included into the input feature set and tuned for optimum sensitivity and specificity in [38]. However, it should be noted that in such approach, the reference information is modified to achieve the best classification performance of the automated method tested, which seems to be rather doubtful.

In order to avoid short false positive AF episodes or short artifact of classified AF the post-processing correction was applied, like dedicated mechanism called AF alarm enhancer [16]. It is the hysteresis counter that begins (or ends) an episode if established number of consecutive analyzed RR segments have been classified as AF (or non-AF). Other post-processing method was based on median filtering [45].

In [16], after combining R-R interval Markov score with two P-wave measurements: the location expressed by P-R interval duration, and the morphology defined as similarity between two consecutive P-waves, the sensitivity did not change, whereas specificity and positive predictive values increased slightly. 

A novel deep learning has been adopted for automated detection of AF in the long-term ECG recordings. This classifier learns directly from the RR intervals and therefore there is no need to extract the features. The model based on deep Recurrent Neural Network (RNN) with Long Short-Term Memory (LSTM) was used in [46], and combining with the Convolutional- and Recurrent-Neural Networks to extract high level features was proposed in [45]. Although a high classification performance has been reported, the computational complexity of deep learning model is much higher than traditional feature-based classifier. In this paper, we describe the method for automated AF detection which assigns the vector of parameters quantitatively describing the HR signal into two classes representing the absence or presence of atrial fibrillation. As estimation of HR variability is also important part of the Fetal Heart Rate (FHR) analysis [47,48,49], the indices widely used for FHR variability description have been considered as potentially useful for AF detection. The detection method presented in this paper was derived from the machine learning principles. Our classification routine was performed by means of the Lagrangian Support Vector Machine (LSVM) [50]—the state-of-the-art classifier based on the linearly convergent learning algorithm. The efficient LSVM learning procedure was obtained from the reformulation of the Quadratic Programing (QP) optimization problem of the Support Vector Machine (SVM) [51]. Additional aggregation stage has been applied to provide more reliable information on risk for the patient. The performance of the proposed AF detection method was examined using the MIT-BIH Atrial Fibrillation database, which includes 25 ten-hour long ECG recordings.

## 2. Materials and Methods

Automated detection of the atrial fibrillation episodes proposed in this work starts with extraction of sixteen HR irregularity features composing the classifier input vector. Then the LSVM classifier is applied to mark a given heartbeat as AF or non-AF one. Final step is aggregation of the classified beats into AF episodes.

### 2.1. HR Irregularity Features

Considering on-line detection of AF and limited computational power of the developed mobile monitor, we applied a simple linear classifier which recognizes the AF heartbeats basing on easily accessible information about heart rhythm and HR features [52,53]. Apart from the HR value, other four input features have been selected in a series of preliminary investigations carried out among larger feature set [54]. Having the information on heartbeats detected, the instantaneous heart rate values HR*_i_* (expressed in beats per minute) are calculated according to the formula:

(1) $${\mathrm{HR}}_{i}\left[\mathrm{bpm}\right]=\frac{60000}{{\mathrm{RR}}_{i}\left[\mathrm{ms}\right]},$$

where: RR*_i_* is the *i*-th interval between two consecutive heart beats expressed in milliseconds.

Next, the features are determined in symmetrical moving window comprising 21 of HR_i_ values:

- MED*_i_* = median{HR*_i−k_*,…,HR*_i+k_*};
- MAD*_i_* = median{x*_i−k_*,…,x*_i+k_*}, where x*i* = |HR*_i_*−MED*_i_*|;
- QNT*_i_*—represents the quantile of order 0.7 estimated over 21 values of heart rate;
- PRP*_i_*—is the ratio of number of HR values between thresholds level of 120 to 160 bpm, to total number.

where: *i* is the number of consecutive heartbeats to be classified, and *k* = 1 … 10.

The values of the additional parameters: window width *N* set to 21, quantile order set to 0.7 and HR thresholds of 120 and 160 were determined as a result of previously performed experiments [54].

For the new classification method, the input vector has been significantly expanded. It additionally comprises seven measures obtained from classical analysis of HR variability used in adults’ electrocardiography. This analysis includes exclusively sinus excitation, i.e., generated by the sinus-atrial node. Thus, it concerns only sinus rhythm variability, and any other types of excitation are excluded and replaced with artificially generated beats. Corrected in this way the series of changes in the subsequent RR intervals become the basis for the determination of heart rate variability measures. The most commonly used quantitative analysis methods can be divided into time, frequency, time-frequency and non-linear methods. In the presented work, the indices describing the HR variability were used in an unusual way as a set of features allowing the detection of atrial fibrillation episodes. The four selected features, obtained in statistical analysis in time domain within the same moving window, are as follows:

- The mean heart rate:
  (2) $$\bar{\mathrm{HR}}\left[\mathrm{bpm}\right]=\frac{1}{N}\overset{N}{\underset{i=1}{\sum }}{\mathrm{HR}}_{i}.$$
- Standard deviation of instantaneous heart rate values:
  (3) $$\text{STD\_HR}\;\left[\mathrm{bpm}\right]=\sqrt{\frac{1}{N-1}\overset{N}{\underset{i=1}{\sum }}{\left(\bar{\mathrm{HR}}-{\mathrm{HR}}_{i}\right)}^{2}}.$$
- Root Mean Square of Successive Differences (RMSSD) which measures the variability within a data set—RR intervals—according to the following equation:
  (4) $$\mathrm{RMSSD}\left[\mathrm{ms}\right]=\sqrt{\frac{1}{N-1}\overset{N-1}{\underset{i=1}{\sum }}{\left({\mathrm{RR}}_{i+1}-{\mathrm{RR}}_{i}\right)}^{2}}.$$
- Percentage of differences between the RR intervals that exceed the value of 50 ms, denoted as pNN50 [%]:
  (5) $$\mathrm{pNN}50=\frac{\sum_{i=1}^{N-1}A_{i}}{N-1}*100\%,$$
  where:
  (6) $$A_{i}=\{\begin{array}{ll} 1 & \mathrm{when}\;\left|{\mathrm{RR}}_{i+1}-{\mathrm{RR}}_{i}\right|>50\\ 0 & \mathrm{when}\;\left|{\mathrm{RR}}_{i+1}-{\mathrm{RR}}_{i}\right|\leq 50 \end{array}$$

In addition, three non-linear features of HRV analysis were applied in the form of:

- Poincare graph, which is a graphical representation of the current interval RR*_i_* plotted against subsequent one RR*_i+1_*. Using the ellipse fitting technique, in each moving window comprising 21 heartbeats, two standard deviations are determined from the points: perpendicular to the regression line (SD1) and along the line (SD2). The SD1 describes the short-term variability of the heart rhythm, while the SD2 refers to the long-term HR variability.
- Turning Points Ratio (TPR) measures the randomness of fluctuations within a data set, by calculating the ratio of the number of turning points to the maximum number of possible turning points. Turning point is found if both the preceding and succeeding points are either greater or lower. It is expected in random data set of arbitrary length *N*, that the number of possible turning points is (2*N* − 4)/3, with a standard deviation of $\sqrt{\left(16N-29\right)/90}$.

**Figure 3:**
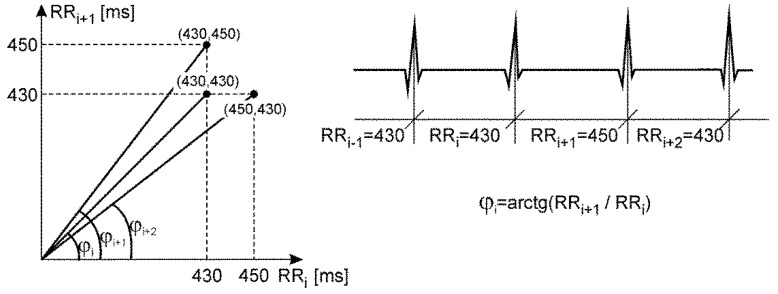
Distribution of successive RR*_i_* intervals in the polar coordinate system, illustrating the definitions of the de Haan’s index describing the short-term HR variability.

A separate group of features used for the detection of AF episodes are parameters commonly used in fetal heart rate analysis [55,56]. It turns out that in perinatal medicine quite different features are used to describe the FHR variability, mainly short-term (beat-to-beat) [57]. For the detection of AF episodes, four widely known short-term coefficients (indices) have been selected [58,59]. They are characterized by high sensitivity to changes in subsequent values of RR intervals and thus they potentially may be useful for AF detection [60,61,62]:

- The Yeh’s index (DI_Yeh) whose determination starts with calculation of the auxiliary values d_i_ representing the ratio of the difference between two successive RR intervals to their sum:
  (7) $$d_{i}=\frac{{\mathrm{RR}}_{i}-{\mathrm{RR}}_{i+1}}{{\mathrm{RR}}_{i}+{\mathrm{RR}}_{i+1}}.$$
  Then, for the analyzed signal fragment, the DI_Yeh index is defined as the standard deviation from the obtained coefficients d_i_:
  (8) $$\text{DI\_Yeh}\;\left[\mathrm{ms}\right]=\sqrt{\frac{1}{N-2}\overset{N-1}{\underset{i=1}{\sum }}{\left(d_{i}-\bar{d}\right)}^{2}},$$
  where: $\bar{d}=\frac{1}{N-1}\overset{N-1}{\underset{i=1}{\sum }}d_{i}$, *N*—number of beats set to 21.
- The Zugaib’s variability index (STV_Zug) has been defined as an average of the absolute values of the differences between successive D_i_ values and their median value:
  (9) $$\text{STV\_Zug}\left[\mathrm{ms}\right]=\frac{1}{N-1}\overset{N-1}{\underset{i=1}{\sum }}\left|D_{i}-\mathrm{Med}\right|,$$
  where: Med—median value for the D*_i_* series, *N*—number of beats set to 21. The D_i_ value represents the ratio of the absolute value of the difference between the heart intervals RR to their sum:
  (10) $$D_{i}=\frac{\left|{\mathrm{RR}}_{i+1}-{\mathrm{RR}}_{i}\right|}{{\mathrm{RR}}_{i+1}+{\mathrm{RR}}_{i}},$$
- The Huey’s index (STV_Huey) was defined as the sum of absolute values of differences of subsequent instantaneous HR values for which the sign of difference was changed:
  (11) $$\text{STV\_Huey}\;\left[\mathrm{bpm}\right]=\overset{N-1}{\underset{i=2}{\sum }}k\cdot \left|{\mathrm{HR}}_{i+1}-{\mathrm{HR}}_{i}\right|.$$
  where:
  (12) $$k=\{\begin{array}{ll} 1 & \mathrm{for}\;({\mathrm{HR}}_{i-1}-{\mathrm{HR}}_{i})\cdot ({\mathrm{HR}}_{i}-{\mathrm{HR}}_{i+1})<0\\ 0 & \mathrm{for}\;({\mathrm{HR}}_{i-1}-{\mathrm{HR}}_{i})\cdot ({\mathrm{HR}}_{i}-{\mathrm{HR}}_{i+1})\geq 0. \end{array}$$
- The definition of de Haan’s index (STI_Haan) is based on a polar coordinate system whose both axes refer to RR intervals expressed in milliseconds, and points represent the pairs of subsequent intervals (RR*_i_*_−1_, RR*_i_*), as shown in Figure 3. STI_Haan is determined as the interquartile range of the angles φ_i_ between the lines connecting the point with origin of the coordinate system, and the X axis, designated for subsequent periods RR*_i_*:
  (13) $$\text{STI\_Haan}=\mathrm{IQR}\left(\varphi_{i}\right),$$
  where: i = 1, 2…*N*,*N*—number of beats.

### 2.2. LSVM Classifier

The proposed method for automated recognition of AF episodes is based on a machine learning approach. To achieve high accuracy of AF detection, we applied the classification routine that originates from the Statistical Learning Theory (SLT) [63]. The SLT is the base for the machine learning methods which are characterized by a high generalization ability, meaning the high efficiency when evaluating previously unknown data i.e., data that have not been used when designing the classifier (also called as classifier training or learning). One of the major achievements of SLT is the Structural Risk Minimization (SRM) principle, which states that the quality of machine learning depends both on the empirical data and the complexity of the model. The most-known practical implementation of the SRM is the Support Vector Machine (SVM) methodology [51,64,65]. The SVM allows for finding the hyperplane in the input feature space which divides the considered classes with the widest margin of separation. The input data that are used to define the margin are called the support vectors. The original SVM algorithm was formulated as a linearly constrained quadratic optimization problem. Consequently, the learning procedure of high computational complexity was obtained [66,67,68]. As the low computational cost of the detection method is of our special interest, in the proposed solution the Lagrangian Support Vector Machine (LSVM) [50] was applied. Its learning replaces the quadratic programming with the linearly convergent iterative algorithm which results in significant reduction of the computational complexity and higher efficiency when compared to the original SVM [50].

Let us consider a training set L, which contains *N*_TRN_ vectors $x_{0}\left(1\right),x_{0}\left(2\right),\;\cdots ,\;x_{0}\left(N_{\mathrm{TRN}}\right)\in R^{t}$ of *t* parameters quantitatively describing the HR signal, and the corresponding output value $y_{0}\left(1\right),y_{0}\left(2\right),\;\cdots ,y_{0}\left(N_{\mathrm{TRN}}\right)\in \left\{-1,\;1\right\}$ defining the absence (non-AF) $y_{0}\left(n\right)=-1$ or the presence $y_{0}\left(n\right)=1$ of the AF episode. The linear SVM classification problem of L can be formulated as the constrained minimization:

(14) $$\underset{R^{N}\times R^{N}}{\min}\;f_{\mathrm{SVM}}\left(w,\xi \right)=\frac{w^{T}\;w}{2}+\gamma \;1^{T}\;\xi ,$$

subject to the condition:

(15) $$D\left(X_{0}w-1w_{0}\right)+\xi \geq 1,$$

and:

(16) ξ≥0,

where $w\in R^{t}$ and $w_{0}\in R$ are the parameters of two bounding planes:

(17) $$\{\begin{array}{l} x^{T}w-w_{0}=+1,\\ x^{T}w-w_{0}=-1. \end{array}$$

separating the training data with the margin $\frac{2}{\left\|w\right\|}$, γ≥0 is a constant that controls the trade-off between model simplicity and model matching to the training data, $1\in R^{N}$ denotes the vector with all entries equal to one, $\xi \in R^{N}$ is the vector of the slack (error) variables, that allow the classes to be bounded with the maximum “soft” margin i.e., with the minimum sum of deviations of training errors and maximum margin for the correctly classified vectors, $D=\mathrm{diag}\left(y_{0}\left(1\right),y_{0}\left(2\right),\;\cdots ,y_{0}\left(N\right)\right)\in R^{N\times N}$ is a diagonal matrix with class labels along its diagonal, $X_{0}={\left[x_{0}^{T}\left(1\right)\vdots x_{0}^{T}\left(2\right)\vdots \;\cdots \vdots \;x_{0}^{T}\left(N\right)\right]}^{T}\in R^{N\times t}$ is the matrix of training input data.

In contrast to SVM, the Lagrange support vector machine maximizes the margin between the separating planes with respect to both orientation (w) and location of the planes ($w_{0}$). Moreover, in the LSVM criterion function the sum of the slack variables $1^{T}\;\xi$ (14) is replaced with the sum of squares $\xi^{T}\xi$ making the constraint (16) redundant. Consequently, the linear LSVM is defined as minimization problem of the functional:

(18) $$\underset{R^{t}\times \;R\;\times \;R^{N}}{\min}\;f_{p\mathrm{LSVM}}\left(w,\;b,\xi \right)=\frac{1}{2}\left(w^{T}\;w+w_{0}^{2}\right)+\frac{\gamma }{2}\;\xi^{T}\xi ,$$

subject to the constraint (17). Moreover, the dual problem of (20):

(19) $$\underset{R_{+}^{N}}{\min}\;f_{d\mathrm{LSVM}}\left(\lambda \right)=\frac{1}{2}\lambda^{T}Q\;\lambda -1^{T}\lambda ,$$

where: $Q=\frac{I}{\gamma }+HH^{T}\in R^{N\times N}$, $H=D\left[X_{0}\vdots -1\right]\in R^{N\times \left(t+1\right)}$ and $I\in R^{N\times N}$ is the identity matrix, has the non-negativity constrain only $\lambda \in R_{+}^{N}$. 

The solution can be determined based on the Karush-Kuhn-Tucker necessary and sufficient optimality conditions [50]. This leads to a linearly convergent iterative scheme which constitutes the LSVM method:

(20) $$\lambda^{\left(k+1\right)}=Q^{-1}\left(1+{\left(Q\;\lambda^{\left(k\right)}-1-\alpha \;\lambda^{\left(k\right)}\right)}_{+}\right),$$

where: *k* is the iteration index and ${\left(Q\;\lambda^{\left(k\right)}-1-\alpha \;\lambda^{\left(k\right)}\right)}_{+}\in R^{N}$ is the vector with all of its negative components set to zero.

The above algorithm is convergent for any starting point if:

(21) $$0<\alpha <\frac{2}{\gamma }.$$

The parameters of the bounding planes that separate the classes can be recovered from the solution of the dual problem by using the following formulas:

(22) $$\{\begin{array}{c} w=X_{0}{}^{T}D\;\lambda ,\\ w_{0}=-1^{T}D\;\lambda ,\\ \xi =\frac{\lambda }{\gamma }. \end{array}$$

The LSVM approach reduces significantly the time necessary to perform calculations for the optimal $\left(w,w_{0}\right)$ while preserving high classification efficiency of the original SVM learning. 

The basic LSVM is a linear classifier, thus to handle the non-linearly separable data the so-called “kernel trick” is required. It is based on the premise that the complex non-linear classification problem will be linearly separable in some feature space of higher dimensionality and involves the non-linear transformation of input data in the high-dimensional space. The linear separating plane $x^{T}w-w_{0}=0$ is then replaced by the non-linear surface:

(23) $$K\left(x_{e}^{T},X_{0e}^{T}\right)\;D\;\lambda =0,$$

where: $x_{e}={\left[x^{T}\vdots -1\right]}^{T}$, $X_{0e}=\left[X_{0}\vdots -1\right]$, and K is the kernel function. Redefinition of the dual problem (19) by using:

(24) $$Q=\frac{I}{\gamma }+DK\left(X_{0e},X_{0e}^{T}\right)D,$$

which makes the LSVM iterative schema (20) valid for any positive semidefinite kernel K [50]. In the proposed approach we used the radial (Gaussian) kernel:

(25) $$K\left(x,y\right)=\exp\left(-\chi \;{\left\|x-y\right\|}^{2}\right),\;\mathrm{where}\;\chi >0.$$

### 2.3. Performance Evaluation

The performance (generalization ability) of the AF classification was evaluated by the classification accuracy (CA), defined as the percentage of correctly classified cases in the testing set (data which was not used during classifier training). As the AF detection process is a kind of diagnostic test giving negative or positive results, we also measured the classification quality using sensitivity (Se), specificity (Sp), positive (PPV) and negative (NPV) predictive value, calculated for the testing data set using a confusion matrix. Since evaluation of the classification efficiency is difficult when analyzing all the prognostic measures simultaneously, we calculated also the F-Score (FS), defined as a harmonic mean of Se and PPV:

(26) $$\mathrm{FS}\;\left[\%\right]=\frac{2\cdot \mathrm{Se}\cdot \mathrm{PPV}}{\mathrm{Se}+\mathrm{PPV}}.$$

### 2.4. Heartbeat Aggregation

The aggregation of the classified heartbeats should lead to removal of accidental changes of heartbeat status, and thus to obtain more reliable information on AF episodes. This process is controlled by two parameters: the window width and percentage threshold. Each heartbeat status is validated in symmetrical window by checking if the number of the heartbeats with the same status exceeds the percentage threshold. We defined the percentage threshold for the AF status, as the number of heartbeats classified as AF to the number of all beats in the analyzed window. If the threshold is exceeded the AF status remains unchanged, otherwise is set to non-AF. The optimal values of the control parameters (window width and the percentage threshold) have been found to ensure the best AF detection performance expressed by the maximal value of the F-Score.

### 2.5. Material

To verify the effectiveness of the proposed AF detection method we have used the MIT-BIH Atrial Fibrillation database (MIT-BIH AF) [4,5], which includes 25 ECG signals, each of 10 h in duration. Of these, 23 ECG signals are accompanied by time markers of detected QRS complexes, while two signals are represented only by information on heart rate, which however is enough for this study. The database contains a total of 1,221,534 heartbeats, with 519,788 annotated as AF. However, when using the window of 21-beat width shifted with one beat, first and last ten heartbeats in each signal were excluded as the HR features were not determined for those heartbeats in incomplete window. For all 25 signals 500 beats were excluded. Finally, our research material consisted of 1,221,034 heartbeats of which 519,664 were related to AF episodes. 

The aim of our research was to achieve the highest quality of classification (maximum FS value) of the all MIT-BIH AF database. In the subsequent experiments we considered each of the *N*_ALL_ = 1,221,034 heartbeats as an independent event. Such a large number of feature vectors to be processed makes the application of LSVM based classifier difficult. For example, the LSVM training requires matrices of *N*_TRN_ x *N*_TRN_ dimension, where *N*_TRN_ is the size of a training set. Hence, when applying only half of the heartbeats from MIT-BIH AF database (*N*_TRN_ = 610,517) for training, only one of these matrices would require approximately 2982 GB of the RAM memory (when stored as double-precision floating-point values). At the same time the efficiency of the LSVM classifier depends to a large extent on the choice of the training data [69]. For this reason, one of the main objectives of our study was to find a training set of the smallest possible size that still would guarantee a satisfactory quality of AF detection. At the first stage, we investigated the ability to distinguish between the AF and non-AF episodes for all data by applying the LSVM classifier trained using heartbeats extracted (drawn randomly) from a single record only. In this way, we were able to specify which signals are most useful for the LSVM classifier training, i.e., leading to the highest classification quality of the all database, as well as to determine the size of the training set necessary to achieve the satisfactory level of the F-Score values. On this basis, we conducted learning by randomly selecting training data from the all database and from a selected group of signals (characterized by the highest values of prognostic measures).

The percentage of the heartbeats marked as AF episodes varies (Table 1). For example, signal 00735 contains only 0.83% of AF episodes (*N*_AF_ = 332), while signals 07162 and 07859 consist exclusively of AF episodes (*N*_AF_ = 39,277 and *N*_AF_ = 60,245, respectively). Except only one signal 06995, there are large disproportions between the numbers of beats representing the AF absence and presence (see *N*_AF_/*N*_SIG_ in Table 1, where *N*_SIG_ is the total number of the heartbeats in a given signal), which may adversely affect the LSVM training [69]. To avoid the problem of poor classification efficiency, being the result of highly imbalanced data, the same number of cases from the minority and the majority class was randomly drawn from a given signal (without replacement) to maintain an equal size of both classes in the training data [70,71,72]. Also, as the generalization ability of a classifier is of crucial importance, only 50% of the heartbeats from the minority class of given signal were used during LSVM training. All the remaining heartbeats (AF and non-AF episodes) were used as a testing set to estimate the classification quality.

In order to explain the way of selecting the training data from a given signal, let us consider an example of a training set that was formed based on heartbeats originating from the signal 00735. As the AF is the minority class in this signal, firstly 166 (50% of the minority class) heartbeats annotated as AF were randomly selected as the training data. Secondly, the selected AF data were completed with 166 heartbeats that were randomly drawn from the non-AF heartbeats of the signal 00735. Finally, these 332 heartbeats were enclosed in the training, while the remaining 1,220,702 in the testing set. From each signal 50 different training sets were generated at random. As there are no non-AF episodes in the signals 07162 and 07859 (Table 1), we could not use the data extracted from these signals for classifier training.

## 3. Results

### 3.1. LSVM Classifier Performance

For the purpose of the LSVM classification the class labels +1 (−1) were assigned to the feature vectors that represent the presence (absence) of the AF episode. The input data were scaled to the range [−1,+1] as recommended in [50]. To guarantee the convergence of the LSVM learning, the parameter α was set to 1.9/γ. The stop condition was an execution of the maximum number of 100 iterations or $\|\lambda^{\left(k\right)}-\;\lambda^{\left(k-1\right)}\|\leq 10^{-5}$.

The Monte Carlo Cross Validation procedure was used to assess the classification performance [70,73]. In each experiment, the MIT-BIH AF database was 50 times randomly divided into separate training and testing sets. The mean values and standard deviations of performance measures for all 50 trails are presented as the final results. 

At the beginning five training sets chosen from the five signals (25 training sets in total) of the smallest size, i.e., data extracted from signals 05091, 00735, 06453, 04015, and 04048, were used to find LSVM classifier parameters *γ* and *χ*. Their values were searched within the set $\left\{10^{-5},\;4\cdot 10^{-5},7\cdot 10^{-5},10^{-4},4\cdot 10^{-4},\;7\cdot 10^{-4},\cdots ,7\cdot 10^{4},10^{5}\right\}$. Parameters providing the highest mean F-Score calculated for all the 25 testing sets (*γ* = 10, and *χ* = 4) were selected and used in all numerical experiments performed.

Table 2 shows the results of classification of testing data using the training sets which were extracted separately from each of the available signals. One can notice that the highest classification sensitivity of the classification Se = 99.12 ± 0.29% was obtained using the training data extracted from the signal 05091, and the highest specificity Sp = 97.42 ± 0.17% by using the training data selected from the signal 04126. However, the highest classification quality FS = 93.39 ± 0.22%, as well as CA = 94.22 ± 0.19%, were obtained with the training data extracted from the signal 08405.

The best FS does not apply to the highest relative size of the training set (*N*_TRN_/*N*_ALL_ = 2.25%, signal 06995), but to the signal 08405 (*N*_TRN_/*N*_ALL_ = 1.13%), where only 5.72% of the heartbeats (on average) from the all database were classified incorrectly. Not the size of the training set is of crucial importance, but the occurrence of the input vectors containing the quantitative parameters of HR variability description which allow for separating the AF and non-AF heartbeats with the widest separation margin (support vectors), guarantying the best classification quality.

To allow a comparison with the results reported in the literature, in the last column of the Table 2 values of the FS_ALL_ are presented. They were calculated by using all heartbeats from the MIT-BIH AF database (*N*_ALL_ = 1,221,034). It is necessary to emphasize that the positive bias of the classifier efficiency being the result of incorporating the classification results of the training data, is insignificant here as the mean difference between FS_ALL_ and FS is equal to 0.09%. It is due to the very small size of the training data up to a maximum of 2.25% of the size of the MIT-BIH AF database (signal 06995, see Table 2, column 2).

During our next experiment, we investigated the LSVM generalization ability when training with balanced (of an equal size of AF and non-AF classes) data sets extracted from the mixed signals. The maximum size of training data for that experiment was determined basing on analysis of performance measures obtained for particular signals, as listed in Table 2. We have assumed satisfactory classification quality as FS > 90%. Since the effectiveness of the LSVM classifier increases with the number of training data, among training data of different size which ensured FS > 90%, we selected the maximum size being equal to 1.39% of total number of heartbeats (signal 04746). It refers to 16,979 heartbeats (see Table 1). Finally, 17,000 training vectors were randomly selected from each of the following signals set:

- mixed two signals of the highest FS (08405) and Se (05091)—marked as the Training Data no. 2—TD_2_,
- TD_2_ with additional signal of the highest Sp (04126)—TD_3_,
- all the signals—TD_4_.

In each of these cases the classification performance was calculated, as in previous experiments, for 50 different training/testing data divisions. The obtained classification results are presented in Table 3. As reference, the classification results using the training data extracted from the signal 08405 (TD_1_), providing the highest FS value during the previous experiment, are shown as well.

The balanced training vectors (TD_2_, TD_3_), being extracted from those signals that provided the best quality of the AF detection during our previous experiment, did not improve the classification efficiency. In fact, lower classification quality was obtained when comparing to the classification results based on training data extracted from the signal 08405 only (TD_1_). However, by applying the training data that was randomly drawn from all signals (TD_4_) we achieved the highest quality FS = 97.26 ± 0.04% (FS_ALL_ = 97.30 ± 0.04%), and the highest accuracy CA = 97.42 ± 0.04% (CA_ALL_ = 97.44 ± 0.04%). It is worth to emphasize that we were able to get these results using only 1.39% of all MIT-BIH AF heartbeats as the training data. This confirms very high classifier generalization ability. The highest FS (FS_ALL_) value that has been noted among the fifty various divisions was equal to 97.34% (97.38%).

One of the basic goals of our work was also to verify if the application of the fetal heart rate variability features, apart from the classical parameters of ECG signal variability, improves the automated recognition of AF episodes. Hence, we investigated how the quality of LSVM classification is affected by the exclusion of FHR variability parameters from the analyzed feature vectors. Table 4 shows the results of the AF classification after removing Yeh’s, Zugaib’s, Huey’s and de Haan’s indices, while maintaining the same divisions of the research data into training and testing sets.

Similarly, as in previous experiments, we assessed both the generalization ability of the LSVM classifier (estimated based on testing sets only) and the classification quality of the all MIT-BIH database. It may be observed that the absence of the FHR variability features resulted in a lower quality of classification. The mean value of the difference between the FS (FS_ALL_) values calculated for all considered training data was equal to 1.15 (1.14) percentage point, with the minimum 0.27 (0.27) for TD_4_ and the maximum 2.21 (2.18) for TD_1_. We can conclude from these results that the introduction of the FHR variability features improves the quality of automated detection of AF episodes based on the LSVM classifier.

### 3.2. Optimal Beats Aggregation

**Figure 4:**
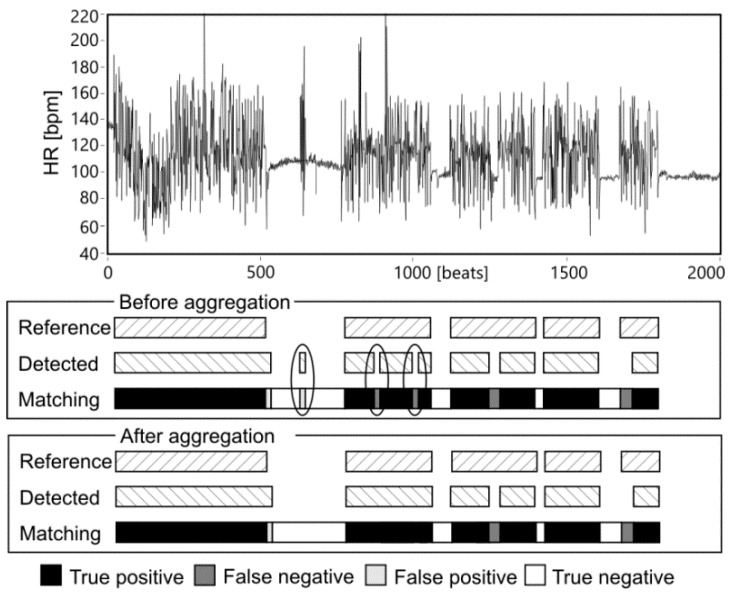
An example of matching the reference and detected AF episodes before and after aggregation. Accidental changes of AF status eliminated in the aggregation stage are marked.

**Figure 5:**
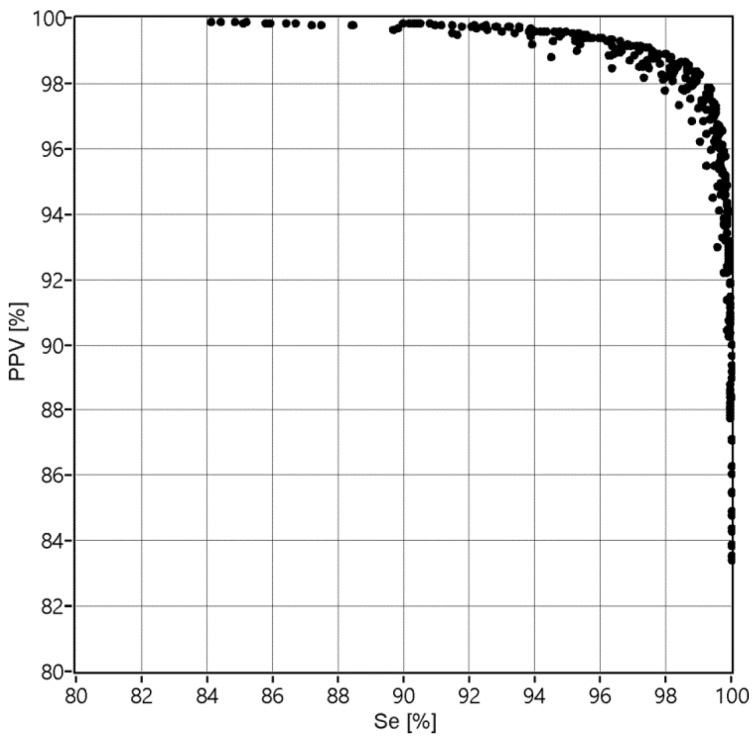
The values of Se and PPV obtained for 361 combinations of the applied validation criteria: window width w = {10, 20,…,190} and percentage threshold p = {5, 10,…, 95}.

The results obtained after LSVM classification (for division of the higher FS_ALL_ = 97.38%, that has been noted among the fifty various divisions in the training data TD_4_) were used for final AF detection—aggregation of classified heartbeats into AF episodes. Reference AF episodes and the detected ones are expected to overlap each other, but usually they overlapped partially, leading to the cases shown in Figure 4. The TP, TN, FP and FN cases were used to calculate the values of Se, PPV and FS during the process of finding the optimal parameters of the validation window width and the percentage threshold. During that process the window width was changed from 10 to 190 beats with step of 10 beats, while the threshold from 5 to 95% with 5% step. Figure 5 presents how considered values affected the Se and PPV, where each of 361 points refers to a given pair of window width and threshold values.

**Figure 6:**
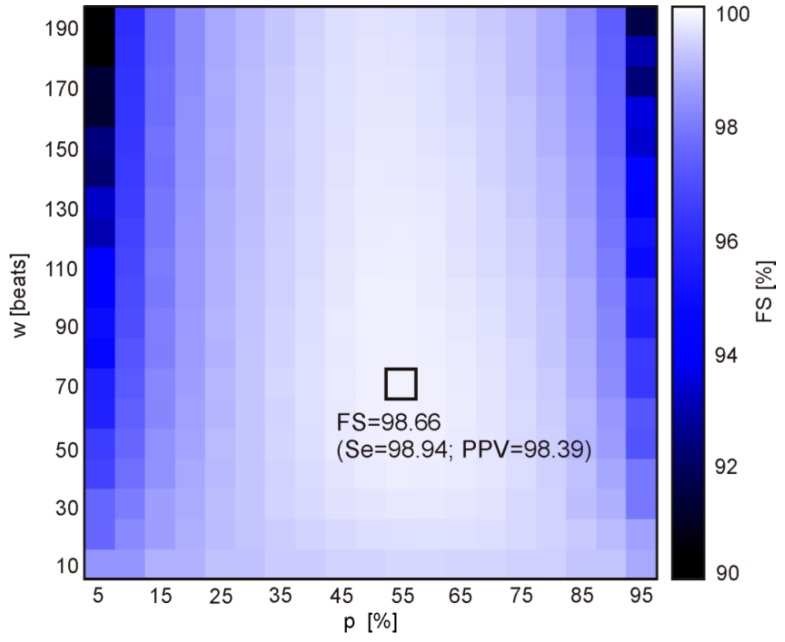
The distances of points defined by (Se(*w*, *p*), PPV(*w*, *p*)) to the point of the highest values (100%, 100%), obtained for the 361 combinations of the following validation criteria: window width *w* = {10, 20,…,190} and percentage threshold *p* = {5, 10,…, 95}. The brightest rectangle indicates the maximum FS (the highest algorithm performance) determined for a given combination (*w*, *p*).

The best performance expressed by the maximum value FS_max_ = 98.66%, relating to the Se = 98.94% and PPV = 98.39%, was obtained for the window width of 70 beats and the percentage threshold of 55%. It can be noted from Figure 6 that the performance increases with an increase of the threshold percentage from 5 to 55%, and then that control parameter does not affect the performance anymore. Considering the number of heartbeats to be aggregated, it is clear that the window should comprise at least 50 beats. Increasing the window width above this value causes slight improvement of the performance, up to optimal value of 70 heartbeats.

### 3.3. AF Detection Performance

The values of the performance measures: Se, Sp, PPV, NPV, CA and FS have been calculated with and without aggregation stage (Table 5). All the performance measures increased after optimized aggregation.

## 4. Discussion

The method for automated detection of the episodes of atrial fibrillation in long-term ECG records has been described in this paper, that represents a new approach derived from the machine learning principles—the Lagrangian Support Vector Machine (LSVM). The performance of the proposed method was evaluated on the MIT-BIH Atrial Fibrillation database, which has already been widely used enabling to compare our results with those reported earlier. On this research material the LSVM classifier, fed with sixteen features describing the heart rate variability, ensured the sensitivity 98.10%, specificity 97.50%, positive predictive value 96.67%, classification accuracy 97.75 and F-Score 97.38%. After aggregation stage those performance measures increased to 98.94%, 98.80%, 98.39%, 98.86% and 98.66 respectively. Especially, significant increase of the PPV value was noted a lower number of false AF detections. Obtained performance is higher than that provided by previously developed classification method based on linear classifier, where Se = 95.42% and PPV = 94.97% [53]. Thus, the proposed more advanced method has better ability to detect the true occurrences of AF and provides lower number of false arrhythmias. It should be emphasized that both in case of simple linear classifier and advanced LSVM approach the aggregation stage significantly improves the efficiency of AF episodes detection. During this study the HR features have been determined in 21 beats wide window [54]. Nevertheless, the results obtained so far by the authors are better than those provided by other automated AF detection methods reported earlier, evaluated using the MIT-BIH AF database (Table 6).

The most obvious feature to measure the RR irregularity seems to be the difference between successive intervals RR. In [33] the standard density histogram of RR differences was prepared as a template—using the annotated AF episodes, and then the similarities between the density histograms of the test data and the standard density histogram were estimated using the standard coefficient of variation (CV test) and Kolmogorov-Smirnov (K-S) statistical test. For the optimal threshold of the test output found by ROC, the K-S test showed Se = 94.4%, Sp = 97.2%, and PPV = 96.1%, for the window length of 100 intervals. Detection of AF episodes based on density histogram of RR differences was also developed by Huang et al. [74]. The proposed more advanced analysis included two steps: AF event detection using the delta RR interval distribution difference curve and AF event classification. Using the ROC curves for determining the threshold of the K-S test, the authors have achieved the higher Se and Sp (96.1% and 98.1%, respectively) for the MIT-BIH AF database. The algorithm described in [34] has been based on the extraction of simple geometric features determined from the histogram of RR prematurity, computed as the percentage variation from the current heart rate and the differences between two successive RR intervals. The feature set included: number of nonempty bins, main distribution width, difference between mean and median and geometric test of bimodality. The score system was introduced to finally classify ten-second segment as non-AF or AF period. Using the MIT-BIH AF database, the RR prematurity algorithm provided the sensitivity of 91% and PPV of 92%, while for the RR differences Se = 92%, and PPV= 78%. The map that plots RR intervals versus RR differences was proposed in [35]. For reference, a window was labeled as true AF episode if 1/2 of intervals in the window were annotated as AF. Threshold value of discriminative parameter—nonempty cell—was determined by ROC, and led to sensitivity 95.8% and specificity 96.4% for the optimal window length of 128 intervals. 

Another linear transformation of RR intervals to differentiate between AF episodes and normal sinus rhythm was described in [44]. The proposed algorithm starts with preprocessing (estimating the RR trend and filtering the ectopic beats), then two functions to measure the RR irregularity are calculated, and finally fusion of these signals is used for detection of AF episodes relying on the fixed threshold. Based on the distribution of the fusion signal output for AF and non-AF beats, the optimal detection threshold with identical values of sensitivity and specificity was set. Using the MIT-BIH AF the authors reported very high values of sensitivity (97.1%) and specificity (98.3%). For that approach a very short window of 8 beats was found as optimal. The authors underline that the proposed method can be matched to detect very short episodes, but it is at the expense of lower specificity. 

The next approach applying only RR interval was based on the variance of normalized RR intervals over ten-second sliding window [29]. According to the authors, the normalization improves the detection performance. The authors used the morphology independent QRS detector to compute RR intervals and variance, and then they smoothed the resulting classifications, using simple majority voting scheme over 600 beat windows, for further robustness. However, the tests carried out on the MIT-BIH AF database showed that the proposed algorithm has sensitivity of 96% but specificity only of 89%, which is sufficient for AF screening only. The more advanced normalization of RR intervals by an affine transformation was proposed in [30]. Interval irregularity was represented by the sparseness of normalized interval probability distribution which was measured by the normalized entropy calculated in the window. The authors used three lengths of the window (30, 50 and 70 beats) to show their influence on the normalization. The ROC analysis enabled them to find the threshold value for the entropy classifier output, that ensured the following values of Se, Sp, PPV and CA: 96.39%, 96.38%, 95.19%, 96.38%.

The sequence of RR interval is assumed to be controlled by a stationary first-order Markov process characterized by a transition probability matrix as it was proposed for the first time by Moody and Mark [4] for automated detection of the AF episodes. As Markov score reflects the relative likelihood of RR intervals sequence in AF episode versus no-AF one, it can be compared to the fixed threshold applied to classify the sequences [16]. In that work the duration statistics with combining all records into one provided the values of Se, Sp, and PPV: 94%, 98% and 97%. Furthermore, a possibility of improvement of the AF episode detection by additional information on ECG morphology was investigated. When the RR interval Markov score was completed with the two P-wave measurements: the location (P-R interval variation) and the morphology (similarity between two consecutive P-waves) the Sp and PPV increased to 99%, while the Se remained unchanged. As the author concluded reduction of the false positive cases is a result of detecting valid P-waves on the ECG recording with irregular rhythm other than AF. Nevertheless, the sensitivity which defines the ability to detect true AF occurrences was significantly lower than the value achieved by our method.

In [37] the changes in RR duration during the sequence have been represented as the binary words, where value of 1 corresponds to increase of interval duration, and 0 means no change or a decrease. Then, the testing segment is classified by comparing its information-based dissimilarity index with those obtained for the templates of AF episode and normal sinus rhythm. Parameters of the classification model: the number of bits, window length and the shift for the dissimilarity comparison boundary were optimized to provide the best performance expressed by sensitivity of 97.04%, specificity of 97.96% and classification accuracy of 97.78%. Another approach based on mapping the RR sequence into symbolic one was proposed in [36]. The detection proceeds in three stages: the initial, where a RR interval sequence is pre-processed with nonlinear and integer filters, the second, where the information of the RR interval changes is converted into symbolic sequence, and final, where the Shannon entropy is calculated to discriminate whether or not the sequence relates to AF episode. Optimal discrimination threshold of Shannon Entropy was obtained by ROC analysis. The RR sequences of 127 beats were processed. The following value of Se, Sp, PPV and CA were: 96.89% 98.25% 97.62% 97.67%, while for the online version of the algorithm: 97.37%, 98.44%, 97.89% and 97.99% [75]. 

The entropy concept, referring to the disorder or uncertainty of a process, was used in many methods for automated detection of AF episodes, usually being included in the feature set, but also as the only measure of RR irregularity. Three statistics describing randomness, variability and complexity of the RR interval time series were proposed in [38]. The turning points ratio, root mean square of successive RR differences and Shannon entropy were employed to characterize the atrial fibrillation. Using the thresholds and data segment of 128 beats determined by ROC the sensitivity of 94.4% and specificity of 95.1% were achieved for the signals from the MIT-BIH Atrial Fibrillation Database. The optimized sample entropy measure, called coefficient of sample entropy (CoSEn), being able to detect very short AF episodes (even 12 beats) was proposed by Lake and Moorman [15]. This feature estimated the probability that short templates will match with other segments within the analyzed RR interval time series. That process was controlled by two parameters: the template length and the tolerance matching, whose optimal values were established by ROC analysis. The authors found the cutoff CoSEn value, which differentiate between AF and normal sinus rhythms, to provide a sensitivity of 91% and a specificity of 94%. In [32] the CoSEn was combined with three other features: the coefficient of variance, root mean square of the successive differences, and median absolute deviation. The detection performance of each irregularity measure was assessed individually by ROC analysis, and CoSEn performed best. The above parameters were also used as the input features set for two classifiers: random forest (RF) and k-nearest neighbor. Both classification models significantly improved the Sp and PPV values over CoSEn, but with substantial drop in Se. The best specificity of 98.3% and PPV of 92.1% were provided by RF model, while the sensitivity achieved the best value 97.6% when using CoSEn as the only discriminative feature. Those results were reported for the combined database, with MIT-BIH AF among others. When only MIT-BIH AF database was employed, the authors noticed significant reduction of detection performance for CoSEn and median absolute deviation, expressed by smaller area under the ROC curve.

Three entropy features: sample entropy, coefficient of sample entropy, Shannon entropy, together with two linear measures: root mean square and normalized root mean square of successive differences constituted the set of RR irregularity measures being tested in [39]. Apart from that HR approach, the authors investigated the ECG-driven approach with two features: peak-to-average power ratio and log-energy entropy, extracted from 2-level stationary wavelet transform coefficients. The support vector machine was used for classification in both approaches. Three different segment lengths were evaluated: 60, 100, 300 beats for HR and 10, 15, 30 s for ECG data. Like in [35], any segment containing at least of 50% AF beats was labeled as true AF when processing ECG. For HR approach this level was reduced to 30%. The longest windows provided the best results for both HR (Se 96.81%, Sp 96.20%, CA 96.45%) and ECG (94.27%, 98.84%, 96.98%, respectively) approaches.

The performance of AF detection using the features extracted exclusively from ECG signals was assessed by Kumar [76]. As the data was taken from MIT-BIH database, the obtained results may be related to those provided by the HR-based methods described here. The proposed classification method employed two features: the log-energy entropy and permutation entropy computed from the sub-band signals obtained using flexible analytic wavelet transform. Using random forest classifier, the authors reported sensitivity of 95.8%, specificity of 97.8% and accuracy of 96.8%.

Two features: the average of RR differences in a defined duration, and the standard deviation of differences in a defined duration, were examined as the inputs of the classifier based on SVM with radial basis function in [40]. The proposed method showed following performance on the MIT-BIH AF database: Se = 95.81%, Sp = 98.44% and CA = 97.50%. The same SVM classifier was employed in [41]. The input set comprised more RR interval features: median heart rate, minimum RR interval, mean RR interval, various entropy measures, and difference irregularity measure. The MIT-BIH AF database was used in that study, but only during the training stage, when very good results have been achieved (sensitivity = 99.07%, PPV = 98.27%, accuracy = 98.84). When testing on a series of 200 signals from the MIT-BIH Arrhythmia database, the best accuracy was 86.60% for the window of 30 beats, sensitivity reached 99.20%, but PPV was only 59.33%. 

The newest approach to automated AF detection proposed in [46] and [45] has been based on deep learning algorithm, which aims to develop the classification model by using all available information from the input. In case of AF detection from the ECG signals it means no need for extraction of the feature neither from raw ECG nor from RR interval time series. In those works the RR data from MIT-BIH AF were partitioned using sliding window of 100 beats [46] or 31 beats but shifted with 10 beats [45], and then fed to Recurrent Neural Network with Long Short-Term Memory. In both works very good results were reported: Se = 98.51%, Sp = 98.32%, CA = 98.67% in [46], and 98.98%, 96.95%, 97.80% with PPV of 95.76% in [45], when median filtering was used as post processing to improve the detection performance. It should be noted, however that development of the deep learning algorithm has been enabled by recent advances in parallel computing on Graphics Processing Units. The computational complexity of deep learning model is much higher than traditional feature-based classifier. This limits its application in wearable devices for long term monitoring with online AF detection, like wristband monitor in a form of wrist bracelet.

## 5. Conclusions

Despite serious medical consequences, atrial fibrillation is still an underestimated clinical and diagnostic problem. Recognition of this form of arrhythmia requires a long-term monitoring of the heart rhythm, since very often patients are asymptomatic. Moreover, the AF episodes can occur accidentally and may last from minutes to hours. The objectivity and efficiency of the visual analysis of long-term recordings can be improved by automated AF detection.

The paper proposed a LSVM-based approach with an original training stage which outperforms other automated AF detection methods based on the information on beat-to-beat irregularity proposed in the literature. Our method ensures a very high efficiency in detection of true AF episodes expressed by sensitivity of 98.94%, and at the same time low number of false episodes, as the positive predictive value reached 98.36%. These results were achieved with post-processing aggregation stage, showing a need for final verification of the classified beats. It also turned out that extending the input feature vector to include parameters describing the heart irregularity and being typically used in the fetal heart rate analysis, had positive effect on classification efficiency. Designing the LSVM-based classifier to deal with such large amount of data like from MIT-BIH AF Database led us to valuable conclusion. Not the size of the training set is of crucial importance, but the occurrence of the input vectors containing the quantitative parameters of HR variability description which allow separating the AF and non-AF heartbeats with the widest separation margin (support vectors), thus guaranteeing the best classification quality.

## Figures and Tables

**Table 1:** The size of the analyzed classes described by the number of AF (*N*_AF_), non-AF (*N*_nAF_) episodes, as well as by the percentage of AF episodes (*N*_AF_) to the total number of the heartbeats (*N*_SIG_) in a given signal.

	**Signal**												
	**00735**	**03665**	**04015**	**04043**	**04048**	**04126**	**04746**	**04908**	**04936**	**05091**	**05121**	**05261**	**06426**
*N_AF_*	332	11057	525	14634	813	3293	30873	5810	39681	138	33759	934	53115
*N_nAF_*	39880	41687	43459	47260	39100	39546	16979	55929	13944	36634	16101	44579	2019
*N_AF_*/*N*_SIG_	0.83%	20.96%	1.19%	23.64%	2.04%	7.69%	64.52%	9.41%	74.00%	0.38%	67.71%	2.05%	96.34%
	**Signal**												
	**06453**	**06995**	**07162**	**07859**	**07879**	**07910**	**08215**	**08219**	**08378**	**08405**	**08434**	**08455**	**All**
*N_AF_*	445	27505	39277	60245	40035	6758	33118	14194	11478	45083	2310	44252	519664
*N_nAF_*	34371	27663	0	0	16538	29820	10217	45078	34016	13752	37519	15279	701370
*N_AF_*/*N*_SIG_	1.28%	49.86%	100.0%	100.0%	70.77%	18.48%	76.42%	23.95%	25.23%	76.63%	5.80%	74.33%	42.56%

**Table 2:** The results of MIT-BIH AF database evaluation using the LSVM classifier (γ=10, χ=4) trained with the balanced data separately extracted from each of the signal. The second column provides the percentage of the size of the training data (*N*_TRN_) to the number of all heartbeats (*N*_ALL_) in the MIT-BIH AF database. Columns 3–8 shows the results for testing data only (not used during the learning phase), the last column presents the F-Score values (FS_ALL_) calculated using all heartbeats from the MIT-BIH AF database. The best results are in boldface.

Signal	*N*_TRN_/*N*_ALL_ [%]	Performance Measure [%]						
		CA	Se	Sp	PPV	NPV	FS	FS_ALL_
00735	0.03	93.60 ± 0.53 *	97.54 ± 0.52	90.68 ± 1.10	88.60 ± 1.15	98.03 ± 0.39	92.85 ± 0.54	92.85 ± 0.54
03665	0.91	81.42 ± 0.42	60.55 ± 1.04	96.84 ± 0.08	93.41 ± 0.13	76.87 ± 0.46	73.47 ± 0.77	73.81 ± 0.76
04015	0.04	76.17 ± 1.63	48.14 ± 4.20	96.93 ± 0.46	92.09 ± 0.82	71.66 ± 1.62	63.12 ± 3.55	63.15 ± 3.55
04043	1.20	91.47 ± 1.93	95.61 ± 1.84	88.41 ± 2.95	85.99 ± 2.99	96.48 ± 1.44	90.52 ± 2.01	90.63 ± 1.98
04048	0.07	89.56 ± 2.69	87.37 ± 7.56	91.18 ± 1.59	88.06 ± 1.46	91.01 ± 4.75	87.53 ± 3.88	87.54 ± 3.88
**04126**	0.27	77.81 ± 1.22	51.32 ± 3.02	**97.42 ± 0.17**	**93.64 ± 0.30**	73.02 ± 1.20	66.25 ± 2.56	66.39 ± 2.54
04746	1.39	91.55 ± 0.15	98.80 ± 0.08	86.20 ± 0.27	84.08 ± 0.26	98.99 ± 0.07	90.85 ± 0.14	90.99 ± 0.14
04908	0.48	92.70 ± 0.72	96.64 ± 0.39	89.79 ± 1.32	87.53 ± 1.42	97.31 ± 0.29	91.85 ± 0.74	91.90 ± 0.74
04936	1.14	87.53 ± 0.51	91.68 ± 0.36	84.47 ± 0.94	81.34 ± 0.91	93.22 ± 0.26	86.20 ± 0.48	86.36 ± 0.48
**05091**	0.01	90.29 ± 0.53	**99.12 ± 0.29**	83.75 ± 1.07	81.89 ± 0.94	**99.23 ± 0.25**	89.68 ± 0.49	89.68 ± 0.49
05121	1.32	87.38 ± 0.66	96.21 ± 0.41	80.87 ± 1.16	78.79 ± 1.02	96.66 ± 0.35	86.63 ± 0.62	86.78 ± 0.61
05261	0.08	83.01 ± 2.89	64.54 ± 7.01	96.70 ± 0.23	93.50 ± 0.49	78.78 ± 3.28	76.17 ± 5.10	76.19 ± 5.09
06426	0.17	91.84 ± 0.60	89.91 ± 0.89	93.26 ± 1.05	90.83 ± 1.27	92.59 ± 0.58	90.36 ± 0.66	90.38 ± 0.66
06453	0.04	92.30 ± 0.83	95.70 ± 2.44	89.78 ± 1.57	87.45 ± 1.55	96.63 ± 1.74	91.36 ± 1.01	91.36 ± 1.01
06995	2.25	91.07 ± 0.61	86.59 ± 1.44	94.37 ± 0.30	91.89 ± 0.41	90.54 ± 0.92	89.15 ± 0.82	89.40 ± 0.80
07879	1.35	91.16 ± 0.27	93.12 ± 0.51	89.72 ± 0.29	86.98 ± 0.32	94.65 ± 0.38	89.95 ± 0.31	90.10 ± 0.31
07910	0.55	88.31 ± 0.87	89.04 ± 1.23	87.77 ± 1.10	84.35 ± 1.23	91.55 ± 0.89	86.63 ± 0.98	86.71 ± 0.97
08215	0.84	89.19 ± 0.46	96.44 ± 1.00	83.84 ± 0.84	81.53 ± 0.72	96.96 ± 0.81	88.35 ± 0.50	88.46 ± 0.49
08219	1.16	85.99 ± 1.11	72.70 ± 2.54	95.80 ± 0.55	92.75 ± 0.90	82.64 ± 1.35	81.49 ± 1.70	81.76 ± 1.67
08378	0.94	91.24 ± 0.27	97.58 ± 0.29	86.56 ± 0.47	84.29 ± 0.45	97.98 ± 0.23	90.45 ± 0.28	90.55 ± 0.27
**08405**	1.13	**94.22 ± 0.19**	96.11 ± 0.47	92.82 ± 0.28	90.81 ± 0.30	97.00 ± 0.35	**93.39 ± 0.22**	**93.47 ± 0.22**
08434	0.19	92.81 ± 0.39	97.37 ± 0.51	89.44 ± 0.64	87.23 ± 0.66	97.87 ± 0.40	92.02 ± 0.42	92.04 ± 0.42
08455	1.25	92.99 ± 0.21	97.73 ± 0.19	89.49 ± 0.39	87.28 ± 0.40	98.16 ± 0.15	92.21 ± 0.22	92.32 ± 0.21

* mean ± standard deviation.

**Table 3:** The results of MIT-BIH AF database evaluation using the LSVM classifier (γ=10, χ=4) trained with the balanced dataset (*N*_TRN_ = 17 000), extracted from: the signal of highest FS (TD_1_), mixed signals of the highest Se and FS (TD_2_), mixed signals that were characterized by the highest Se, Sp and FS (TD_3_), and all the signals (TD_4_). The best results obtained for testing data only and for all data are in boldface.

Training Data Selection	Performance Measure [%]					
	Se	Sp	PPV	NPV	FS	CA
Testing data only						
TD_1_	96.11 ± 0.47 *	92.82 ± 0.28	90.81 ± 0.30	97.00 ± 0.35	93.39 ± 0.22	94.22 ± 0.19
TD_2_	95.63 ± 0.76	92.43 ± 0.29	90.31 ± 0.31	96.64 ± 0.56	92.89 ± 0.36	93.79 ± 0.30
TD_3_	82.53 ± 0.94	95.19 ± 0.17	92.68 ± 0.22	88.08 ± 0.56	87.31 ± 0.53	89.82 ± 0.37
**TD_4_**	**98.12 ± 0.08**	**97.31 ± 0.09**	**96.42 ± 0.11**	**98.60 ± 0.06**	**97.26 ± 0.04**	**97.65 ± 0.04**
All the database						
TD_1_	96.17 ± 0.46	92.89 ± 0.27	90.93 ± 0.30	97.03 ± 0.34	93.47 ± 0.22	94.28 ± 0.19
TD_2_	95.70 ± 0.74	92.52 ± 0.28	90.46 ± 0.31	96.68 ± 0.55	93.00 ± 0.36	93.87 ± 0.29
TD_3_	82.81 ± 0.92	95.25 ± 0.16	92.82 ± 0.21	88.21 ± 0.55	87.53 ± 0.52	89.96 ± 0.37
**TD_4_**	**98.14 ± 0.08**	**97.34 ± 0.09**	**96.47 ± 0.11**	**98.61 ± 0.06**	**97.30 ± 0.04**	**97.68 ± 0.04**

* mean ± standard deviation.

**Table 4:** The results of MIT-BIH AF database evaluation when FHR variability features were excluded from recognition of the AF episodes. The LSVM classifier (γ=10, χ=4) was trained with the balanced dataset (*N*_TRN_ = 17 000), extracted from: the signal of highest FS (TD_1_), mixed signals of the highest Se and FS (TD_2_), mixed signals that were characterized by the highest Se, Sp and FS (TD_3_), and all the signals (TD_4_). The best results obtained for testing data only and for all data are in boldface.

Training Data Selection	Performance Measure [%]					
	Se	Sp	PPV	NPV	FS	CA
Testing data only						
TD_1_	91.04 ± 1.75 *	93.61 ± 0.34	91.34 ± 0.41	93.42 ± 1.20	91.18 ± 0.91	92.52 ± 0.71
TD_2_	91.90 ± 1.30	92.64 ± 0.40	90.21 ± 0.48	93.95 ± 0.91	91.04 ± 0.72	92.32 ± 0.58
TD^3^	82.08 ± 1.13	95.17 ± 0.20	92.62 ± 0.25	87.81 ± 0.67	87.03 ± 0.64	89.61 ± 0.45
**TD_4_**	**97.97 ± 0.09**	**97.02 ± 0.10**	**96.04 ± 0.12**	**98.48 ± 0.07**	**96.99 ± 0.05**	**97.42 ± 0.04**
All the database						
TD_1_	91.16 ± 1.73	93.68 ± 0.34	91.44 ± 0.40	93.48 ± 1.19	91.29 ± 0.90	92.61 ± 0.70
TD_2_	92.03 ± 1.28	92.73 ± 0.39	90.37 ± 0.47	94.02 ± 0.90	91.12 ± 0.71	92.43 ± 0.57
TD_3_	82.37 ± 1.12	95.23 ± 0.19	92.76 ± 0.25	87.95 ± 0.66	87.25 ± 0.63	89.76 ± 0.45
**TD_4_**	**97.99 ± 0.09**	**97.04 ± 0.10**	**96.10 ± 0.12**	**98.49 ± 0.07**	**97.02 ± 0.05**	**97.44 ± 0.04**

* mean ± standard deviation.

**Table 5:** The AF detection performance obtained without and with aggregation process, which was optimized for the window width of 110 beats and percentage threshold equal to 55%, calculated for all 1,221,033 reference annotated heartbeats.

Performance Measures [%]	No Aggregation	With Aggregation
Se	98.10	98.94
Sp	97.50	98.80
PPV	96.67	98.39
NPV	98,57	99,21
CA	97.75	98.86
FS	97.38	98.66

**Table 6:** An overview of published results of existing AF detection methods using the MIT-BIH Atrial Fibrillation Database.

Method	Features	Window	Key Techniques	Results			
				Se	Sp	PPV	CA
Tateno et al. 2001 [33]	RR difference	100 beats	Histogram, Kolmogorov-Smirnov test, ROC.	94.4	97.2	96.1	–
Huang et al. 2011 [74]	RR difference	23 beats	Histogram, SD analysis, Kolmogorov-Smirnov test.	96.1	98.1	–	–
Petrucci et al. 2005 [34]	RR difference, RR prematurity	60 s	Geometric measures of histogram, Score system.	92	–	92	–
Lian et al. 2011 [35]	RR interval, RR difference	128 beats	Mapping RR intervals versus RR differences, thresholds.	95.8	96.4	–	–
Petrenas et al. 2015 [44]	RR interval	8 beats	Thresholds	97.1	98.3	–	–
Logan et al. 2005 [29]	RR interval	600 beats	Variance of normalized RR interval, simple majority voting.	96	89	–	–
Islam et al. 2016 [30]	RR interval	70 beats	Normalization of RR intervals by an affine transformation.	96.39	96.38	95.19	96.38
Babaeizadeh. et al. 2009 [16]	RR interval P-wave measurements	-	Stationary first-order Markov process, decision tree.	94	99	98	–
Zhou et al. 2014 [36]	RR interval	127 beats	Mapping the RR sequence into symbolic one, Shannon entropy, ROC.	96.89	98.25	97.62	97.67
Zhou et al. 2015 [75]	RR interval	127 beats	Online version of [36]	97.37	98.44	97.89	97.99
Cui et al. 2017 [37]	RR interval	150 beats	Mapping the RR sequence into symbolic one, dissimilarity index.	97.04	97.96	–	97.78
Dash et al. 2009 [38]	RR difference	128 beats	Turning points, RMSSDD, Shannon entropy, ROC.	94.4	95.1	–	–
Lake et al. 2011 [15]	RR difference	12 beats	Coefficient of sample entropy (CoSEn), ROC.	91	94	–	–
Kennedy et al. 2016 [32]	RR difference	30 beats	Random forest, k-nearest neighbor.	97.6	98.3	92.1	–
Andersen et al. 2017 [39]	RR interval ECG features	300 beats 30 s	Sample entropy, Shannon entropy, CoSEn, SVM.	96.81 94.27	96.20 98.84	–	96.45 96.68
Kumar et al. 2018 [76]	ECG features	1000 samples	Wavelet transform, Random forest.	95.8	97.8	–	96.8
Nuryani et al. 2015 [40]	RR difference		SVM with radial basis function.	95.81	98.44	–	97.50
Colloca et al. 2013 [41]	RR difference Median HR	30	Entropy, SVM with radial basis function	99.20	-	59.33	86.60
Faust et al. 2018 [46]	-	100	Deep Recurrent Neural Network (RNN) with Long Short-Term Memory (LSTM).	98.51	98.32	–	98.67
Andersen et al. 2019 [45]	-	31	Deep learning combining with the convolutional- and Recurrent-Neural Networks.	98.98	96.95	95.76	97.80
Wrobel et al. 2018 [53]	HR irregularity features	21	Linear classifier	95.42	96.12	94.97	95.62
Proposed method 2019	HR irregularity features	21	LSVM	98.94	98.39	98.86	98.66
